# Tumor biobanks in translational medicine

**DOI:** 10.1186/1479-5876-10-204

**Published:** 2012-10-02

**Authors:** Gerardo Botti, Renato Franco, Monica Cantile, Gennaro Ciliberto, Paolo Antonio Ascierto

**Affiliations:** 1 Istituto Nazionale Tumori Fondazione “G. Pascale”, Napoli, Italy

## Abstract

The concept of tissue banking as a “bio-repository” aimed to collection, storing and distribution of human biological material and clinical information, is emerging as a successful strategy to support clinical and translational research. In particular, Tumor Biobanks represent a key resource for diagnosis, research and experimental therapies, especially for those correlated to clinical application of a new type of medicine known as “intelligent drugs”.

Biobanks are not “spontaneous” collections, but they needs an institutional organization, basically a research unit, whose effectiveness and quality can be guaranteed only if it is carefully organized according to precise and shared rules.

## Background

In recent years, human biological material (e.g. tissues, cells, nucleic acids) obtained through common diagnostic procedures has become an important resource for biomedical research. In oncology, tumor tissue samples represent a precious tool for both clinical and experimental research. These tissues, preserved in optimal conditions, are an essential resource for identifying novel biomarkers for specific therapies [1]. Progress in genome and proteome research has also allowed us to differentiate multifactorial diseases previously considered as a single disorder into more precise diagnostic entities [2]. This achievement provides the basis for developing a more personalized treatment approach, optimizing clinical protocols and improving disease prevention.

However, in order to ascertain the etiology of complex diseases, it is necessary to have access to a wide collection of biological samples with epidemiological, clinical, biological and molecular data from a large number of patients and healthy persons.

The identification of standard operative procedures (SOPs) for tissue sampling, storage and collection of clinical data is an essential step in modern biobank management [3,4]. Sampling and collecting procedures must guarantee both the correct pathological diagnosis as well as the biological integrity of macromolecules (DNA, RNA and proteins) that represent the potential subject of investigation. Recently, thanks to the development of more advanced and sensitive methodologies, attention has shifted from the use of fresh/frozen tissues to those fixed in formalin/paraffin-embedded, both of which have been shown to be reliable sources for molecular analyses [5]. In addition, today’s biobank requires meaningful, relevant performance indicators that take into consideration more than just the amount of material collected. For instance, assessment should also consider the quality of collected biomaterial and the accompanying data, as well as the number and quality of projects that have made use of the material, and the number of resulting publications (also considering the journal impact factor) for which the biobank has been the source of tissue samples.

Biobanking is a new and dynamic discipline that is being undertaken across a wide range of institutions, both public and private. The increasing number of biobanks has helped create networks across institutions and countries, these having developed in order to achieve sufficiently large numbers of tissue samples relating to the same groups of pathologies. Thanks to international cooperation, these collections are allowing the study of rare types of cancers, as well as more common tumours.

Several initiatives have been undertaken that aim to organize collection of material with reliable clinical information in order to guarantee high-quality samples adequate for diagnostics, therapy and research. In Europe, more than 225 biobanks and institutions from over 30 countries that collect samples and pathological/clinical data belong to the *Biobanking and Biomolecular Resources Research Infrastructure* (BBMRI) [6]. The aim of the BBMRI is to act as an interface between biological specimens and clinical data (from patients and European populations) and high-quality biomedical research. In this way, the network aims to enhance the scientific excellence of European biomedical research, increasing the competitiveness of European biotechnology and attracting investment from global pharmaceutical industries and research institutions.

## Competing interests

The authors declare that they have no competing interests.

## Authors’ contributions

All the authors read and approved the final manuscript.
